# Discriminative and predictive validity of risk assessment measures for women incarcerated for serious violent offences in Australia

**DOI:** 10.1080/13218719.2023.2242437

**Published:** 2024-01-08

**Authors:** Nina Papalia, Melanie Simmons, Janet Ruffles, Benjamin Spivak, Ashley Dunne, Rachael Fullam, James R. P. Ogloff

**Affiliations:** Centre for Forensic Behavioural Science, Swinburne University of Technology and Victorian Institute of Forensic Mental Health (Forensicare), Alphington, VIC, Australia

**Keywords:** female prisoners, HCR–20^V3^, level of service, LSI–R:SV, LS/RNR, predictive validity, risk assessment, violence, violent offenders, women

## Abstract

Despite the growing population of women in Australian prisons, limited research has explored whether commonly used risk assessments – predominantly developed and tested on men – are valid for women. We investigated the discriminative and predictive validity of the Level of Service Inventory–Revised: Screening Version (LSI-R:SV), Level of Service/Risk, Need, Responsivity (LS/RNR), and the Historical, Clinical, Risk Management 20–Version 3 (HCR-20^v3^) for Victorian women imprisoned for serious violence (N = 79). The LS/RNR was related to any, violent, and non-violent recidivism, and both the LSI-R:SV and the H-Scale of the HCR-20^v3^ were related to violent recidivism, with the H-Scale demonstrating strong predictive validity for violence. Four LS/RNR needs domains demonstrated discriminative and predictive validity for any and/or violent recidivism (criminal history, family/marital, alcohol/drug problem, antisocial pattern). Findings are locally significant, showing that the LS/RNR and HCR-20^v3^ H-Scale are useful for the prediction and discrimination of recidivism for Australian women incarcerated for serious violence.

Despite comprising a relatively small proportion of the total prison population in Australia, the number of females in prison has increased significantly. Between 2009 and 2019, the number of females in Australian prisons (including all states and territories) increased by 64%, compared to a 45% increase in the number of males in prison (Australian Bureau of Statistics, 2019). This increase has been mirrored in Victoria, where the number of females received into prison each year more than doubled between 2012 and 2018 (Walker et al., 2019). This growth in the female prison population, which has also been observed internationally (Walmsley, 2015), underscores the need to ensure that risk assessment instruments commonly used in Australian correctional settings to assess risk of violence are valid and appropriate for both males and females.

In the context of correctional services, violence risk assessment measures play an important role in directing service provision around rehabilitation needs and the development and delivery of effective intervention and management plans, the ultimate aim being a reduction in dynamic risk factors and likelihood of reoffending and, in turn, increased public safety. In Victoria, such measures form part of the tiered assessment framework used by Corrections Victoria to assess risk for violence and reoffending, identify rehabilitation needs and allocate offenders to treatment programmes. Specifically, the Level of Service Inventory–Revised: Screening Version (LSI–R:SV; Andrews & Bonta, 1998) is used to triage all people in prison upon reception as part of an initial classification process. People screened as medium or high risk for general risk of reoffending are then assessed using the full Level of Service/Risk, Need, Responsivity (LS/RNR; Andrews et al., 2008), which includes static (unchangeable) and dynamic (amenable to change) risk factors related to general recidivism. Individuals are streamed into specialised intervention pathways, including a stream for serious violent offenders. Women directed into this stream are then assessed using the Historical, Clinical, Risk Management 20–Version 3 (HCR–20^V3^; Douglas et al., 2013; Webster et al., 1997) as an offence-specific risk assessment. This measure integrates evidence-based static and dynamic risk factors and provides decision-making guidelines to inform clinical judgement and standardise assessments.

According to a large body of international research, commonly used risk assessment measures, including the LS suite of measures and the HCR–20, have been found to demonstrate roughly comparable levels of predictive validity in the moderate to high range (acknowledging some differences as a function of methodological factors, such as varying follow-up periods, definitions of outcome, and populations; e.g. Campbell et al., 2009; Fazel et al., 2012; Yang et al., 2010). However, just as most risk assessment measures have been developed primarily with male samples, there has been relatively limited validation research exploring the predictive validity of violence risk assessments when used with women. This is particularly true in the Australian context; of 18 studies satisfying the inclusion criteria of a recent systematic review on the predictive validity of risk assessment measures for female offenders, only two examined validity in an Australian female offender sample (Gower et al., 2020).

### Risk assessment for female offenders

The question of whether commonly used risk assessment measures are generalisable to female offenders is underpinned by an ongoing debate about whether such measures are gender-neutral or gender-responsive (de Vogel et al., 2019; Olver & Stockdale, 2022; Salisbury et al. 2016; Yesberg et al., 2015). Some argue that, while there may be differences between male and female offenders, key criminogenic needs or risk factors are generally consistent, irrespective of gender (Rettinger & Andrews, 2010). Others maintain that there are unique risk factors for the onset and maintenance of offending for females and/or gender differences in the relative importance of some violence risk factors (Brennan et al., 2012; Hannah-Moffat, 2009; Salisbury et al., 2016). Examples of risk factors that have been identified as potentially impacting female offenders in a gendered way include: relationship issues, financial issues, self-efficacy, substance use, victimisation and trauma (Blanchette & Brown, 2006; Brennan et al., 2012; Davidson & Chesney-Lind, 2009; Olver et al., 2014; Van Voorhis et al., 2010).

Contributing to the ongoing debate is the mixed evidence that has emerged from the limited research examining the predictive validity of violence risk assessment measures in females. International studies have indicated that the HCR–20^V2^ and HCR–20^V3^ may have some predictive value for females in forensic psychiatric and correctional settings (e.g. Coid et al., 2009; de Vogel et al., 2019; Green et al., 2016; Warren et al., 2018). Indeed, a recent meta-analysis of 12 studies with female samples found small-to-moderate effect sizes for the total HCR–20 score for the prediction of violence (*k* = 6, *N* = 493, *r* = .312, *p* < .001) and recidivism (*k* = 5, *N* = 1470, *r* = .249, *p* < .001; Rossdale et al., 2020). Of the subscales, the Historical scale was the best predictor of any recidivism, demonstrating moderate predictive validity (*k* = 5, *N* = 537, *r* = .389, *p* < .001), while future violence was best predicted by the total HCR–20 score. The authors concluded that the HCR–20 is useful for understanding and assessing violence risk in females but needs to be interpreted and applied with caution given the limited research base. Additionally, of the 12 studies included in the meta-analysis, only two examined predictive validity in a female prison population. No published studies have examined the predictive validity of the HCR–20 in Australian female offending populations, despite regular use of the measure in Australian forensic and correctional settings.

International evidence for the predictive utility of the Level of Service Inventory–Revised (LSI–R; Andrews & Bonta, 1995) and associated LS measures for females is more consistent, with studies indicating that the full LS measures (i.e. the LSI–R, LS/RNR and Level of Service/Case Management Inventory, LS/CMI) perform similarly for males and females, with predictive validity ranging from moderate to high (Andrews et al., 2012; Dyck et al., 2018; Gendreau et al., 2002; Jimenez et al., 2018; Olver et al., 2014; Smith et al., 2009). In the largest meta-analysis of the LS family of measures to date, Olver et al. (2014) found that the predictive validity of the total LS risk/need score in the full LS measures was very similar for both males and females, generating effect sizes of .29 for males and females on general recidivism in 31 samples, but slightly higher effect sizes for males (*r* = .29) than females (*r* = .25) for violent recidivism from six samples. There was, however, some indication that certain factors may have special relevance for female offenders, with the Substance Use and Personal/Emotional subscales demonstrating significantly larger effect sizes for females in the prediction of general recidivism (see also Andrews et al., 2012). This suggests that, while the full LS measures may be valid for both males and females, there may be some differences in how the measures work in terms of predicting violence for females and how some LS subscales apply according to gender. Regarding the screening measure, the LSI–R:SV, comparatively little research regarding its predictive validity has been conducted, with the small research base indicating low to moderate predictive validity amongst females for rearrest (Lowenkamp et al., 2009; McCafferty & Scherer, 2017).

There is very limited research in Australia examining the LS measures among offending females, and the research that is available appears mixed. For instance, a New South Wales study found that, in a sample of incarcerated females, the LSI–R total score demonstrated moderate discriminative validity for reincarceration within two years of release overall (area under the curve, AUC = .69; Watkins, 2011). In contrast, a study examining the predictive validity of the LS/CMI (Andrews et al., 2004) found that, in a small sample of female offenders in Tasmania serving community-based orders, the LS/CMI demonstrated very low predictive validity for the prediction of general reoffending within 12 months of the index offence and did not achieve significance (AUC = .58; Gordon et al., 2015). Of the subscales, only the Companions subscale predicted reoffending, with lower scores indicating a decreased likelihood of reoffending. Although the authors posited that the failure to achieve significance might be due to the small sample of female offenders (*N* = 113) and the low rate of reoffending (15.9%), it does underscore the importance of validating measures across jurisdictions and the need for further research in Australia to determine whether violence risk assessment measures are valid for females. No published studies have examined the predictive validity of the LSI–R:SV in Australian female offending populations.

### Current study

The current study aimed to evaluate the predictive validity of three risk assessment measures for recidivism, and particularly violent recidivism, in a sample of 79 women sentenced to prison in Victoria, Australia for a serious violent offence. The paucity of regional research about the validity of risk assessment measures for women who perpetrate violence means that the study will provide valuable information about the degree to which risk assessment measures that are regularly used by Australian correctional agencies – the LSI–R:SV, the LSR/RNR and the HCR–20^V3^ – are applicable to females. Given potential cross-jurisdictional differences in demographics, it is important that these measures are shown to be sufficiently valid and useful in the local context in which they are used.

## Method

### Design

This study used a pseudo-prospective follow-up design to examine the predictive validity of the LSI–R:SV, LS/RNR and HCR–20^V3^ for adult females in prison convicted of violent offences. The sample was drawn from a larger study examining the validity of risk assessments used by Corrections Victoria for the prediction of serious offending among imprisoned individuals in Victoria. The larger study comprised adults who: (a) received a custodial sentence for a serious violent offence, as defined in Section 3 of the *Corrections Act 1986* (Vic),^1^ between 1 January 2015 and 31 December 2017; and (b) completed a risk assessment or commenced/completed an intervention programme.

Socio-demographic information, risk assessment data, incarceration dates and community corrections/parole orders for the cohort were extracted from Corrections Victoria’s administrative databases. All risk assessment measures were administered and scored directly by Corrections Victoria clinicians (HCR–20^V3^) and accredited operational staff who have undergone LS-specific training (LSI–R:SV, LS/RNR) as part of their routine management of individuals convicted for serious violence offences. The study cohort was subsequently linked to Victoria Police databases to obtain information about post-release community offending through to 31 December 2019.

Ethical approval to conduct the study with a consent waiver was obtained from the Department of Justice and Community Safety (Victoria) Human Research Ethics Committee (JHREC; CF/18/17758) and the Swinburne University Human Research Ethics Committee (SUHREC; ID 896). A waiver of consent was approved by JHREC and SUHREC consistent with Section 2.3.10 of the National Statement on Ethical Conduct in Human Research, on the grounds of low overall risk to participants, the anticipated benefits of the research, the impracticality of obtaining informed consent and strong measures to protect the privacy of participants.

### Sample

Among the 97 adult females convicted for serious violent offences from the parent study, we excluded individuals who: (a) did not have LSI–R:SV, LS/RNR or HCR–20^V3^ assessment data after 1 January 2015; or (b) were not released from their index incarceration (i.e. the incarceration period leading to selection into the study) at the end of the follow-up period (31 December 2019). This resulted in a final study sample of *N* = 79; however, not all women had complete risk assessment data across all three measures.

It was not unusual for individuals to have multiple assessments with the same risk measure, particularly for the LSI–R:SV and LS/RNR. In such instances, we applied a decision rule: (a) for those with at least one assessment with the relevant measure during the index incarceration period, we selected the assessment closest to the release date; or (b) for those whose first assessment with the relevant measure occurred after their index incarceration period, we selected the first assessment (i.e. most proximal to release).

### Measures

#### Level of Service Inventory–Revised: Screening Version (LSI–R:SV)

The LSI–R:SV (Andrews & Bonta, 1998) is an eight-item actuarial screening measure to prioritise cases for further assessment. The items capture seven key risk factors – criminal history (two items), employment, criminal associates, substance abuse, personal/emotional, family, and criminal attitudes. The first six items have a ‘yes/no’ response format, and the last two items are scored ‘0–3’, where ‘0 = a very unsatisfactory situation with a very clear and strong need for improvement’ to ‘3 = a satisfactory situation with no (or little) need for improvement’. A ‘yes’ response on Items 1–6 and a ‘0 or 1’ response on Items 7–8 suggest that the risk factor is present and produces an item score of 1. The items are summed to generate a total score ranging from 0 to 8, where, at the time, scores 0–2 were triaged as low, 3–5 as medium and 6–8 as high risk. Corrections Victoria practice within prisons is that individuals screened as medium or high risk, who have six months or more remaining on their sentence, are referred for further assessment with the full LS/RNR.

#### Level of Service/Risk, Need, Responsivity (LS/RNR)

The LS/RNR (Andrews et al., 2008) is an actuarial assessment developed to estimate risk of recidivism, identify rehabilitation needs and assess the most relevant factors related to supervision and management. The General Risk/Needs section contains 43 items across the central eight criminogenic factors – Criminal History (8 items), Education/Employment (9 items), Family/Marital (4 items), Leisure/Recreation (2 items), Companions (4 items), Alcohol/Drug Problem (8 items), Procriminal Attitude (4 items) and Antisocial Pattern (4 items). Each item generates a score of 0 when absent and 1 when present and are summed to create subscale scores and a total score. Actuarial risk ratings are derived from the total General Risk/Needs score and, at the time, included very low risk (0–4), low risk (5–10), medium risk (11–19), high risk (20–29) and very high risk (30–43). Consistent with Corrections Victoria practice at the time, this study used three risk levels: low (includes very low risk), medium and high risk (includes very high risk).

#### Historical Clinical Risk Management–20 Version 3 (HCR–20^V3^)

The HCR–20 (Webster et al., 1997) is a structured professional judgement assessment comprising 20 risk factors for general violence across three scales: 10 historical static factors, which indicate an individual’s long-term risk level (H-Scale); five dynamic clinical factors, which reflect current/recent correlates of violence (C-Scale); and five dynamic risk management factors, which focus on situational and post-assessment factors that may influence risk (R-Scale). Dynamic risk factors also function as potential targets for violence intervention. The HCR–20^V3^ (Douglas et al., 2013) used in the current study is constitutionally comparable to the HCR–20 with some amendments to item content and the addition of relevance ratings.

Corrections Victoria’s risk assessment protocol directs clinicians to complete the H-Scale of the HCR–20 from file review to identify which women require a more comprehensive violence risk assessment. Women who receive a score of 8 or more (out of 20) on the H-Scale are then assessed using the full HCR–20^V3^. Based on combined interview(s) with the examinee and review of collateral information, clinicians code the presence of risk factors using a three-level nominal scale: N = factor not present or does not apply; P = factor is possibly or partially present; and Y = factor is present. A risk factor may also be ‘omitted’ where there is no reliable information by which to judge its presence. With consideration to the presence and relevance of risk factors, clinicians then provide an overall risk judgement/case prioritisation rating (i.e. low, moderate or high). In accordance with the HCR–20^V3^, clinicians are discouraged from calculating a total ‘score;’ however, for research purposes the items can be summed to provide subscale and total scores (where N = 0, P = 1 and Y = 2). Relevance ratings were not extracted for the purpose of this study.

In this sample, 68.4% (*n* = 54) of women had a full HCR–20^V3^ assessment recorded in their electronic file. Although an overall risk judgement was documented for all these women, item-level data (i.e. H-Scale, C-Scale, R-Scale) were available for 47 of these women (59.5%). There were an additional 18 women who had complete item-level data for the H-Scale (i.e. in the absence of a full HCR–20^V3^ assessment); thus, 82.3% (*n* = 65) of the sample had at least the H-Scale score from the HCR–20^V3^.

#### Recidivism

Victoria Police’s Law Enforcement Assistance Program (LEAP) database was used to extract recidivism data for the study cohort. Any recidivism was defined as a police charge in Victoria for any offence while at risk in the community until the end of the observation period (31 December 2019). We used charges as the measure of recidivism rather than convictions because at court, charges are frequently combined or dropped during progress toward a guilty plea. Offences were further categorised as either violent (nonsexual) recidivism or non-violent recidivism. Charges such as murder, assault, kidnapping, threats (including intents to harm and cause fear), robbery, aggravated burglary, affray and stalking were classified as violent recidivism. Sexual recidivism (e.g. rape, sexual assault, indecent acts and threats to commit a sexual offence), although included in ‘any recidivism’, was not examined separately because the measures were not developed to assess risk of sexual recidivism (*n* = 1 woman had sexual recidivism during the follow-up period). Charges that were neither violent nor sexual in nature (e.g. property-related offences, fraud-related offences, theft, breach offences and drug-related offences) were classified as non-violent recidivism.

### Statistical analysis

Data were analysed through RStudio using R Version 4.1.1 (R Core Team, 2021) with several packages: *tidyverse* (Version 1.1.3; Wickham, 2021), *Hmisc* (Version 4.6-0; Harrell Jr & Dupont, 2021), *psych* (Version 2.1.9; Revelle, 2021), s*urvival* (Version 3.2-11; Therneau et al., 2021)*, pec* (Version 2020.11.17; Gerds, 2020), *timeROC* (Version 0.4; Blanche, 2019), *rms* (Version 6.2-0; Harrell Jr, 2021) and *cutpointr* (Version 1.1.1; Thiele, 2021).

Risk assessment scores extracted from Corrections Victoria’s administrative databases were checked for accuracy by re-computing them from the extracted item-level data. Some minor errors were detected for the extracted HCR–20^V3^ subscale/total scores (e.g. individual item scores were summed incorrectly), and thus researcher-calculated scores were used for all subsequent statistical analyses. Where individual HCR–20^V3^ items were omitted, but the number of omitted items was below a threshold for complete exclusion (≤4 for total score; ≤2 for H-Scale score), pro-rated scores were used consistent with Brookstein et al. (2021).

To determine whether the risk assessment risk-level groups differed in the rate of surviving over time, Kaplan–Meier survival analysis (Kaplan & Meier, 1958) was used. For each recidivism outcome, Kaplan–Meier curves were used to plot the proportion of each risk group that 'survived’ (i.e. did not recidivate) over time. Log-rank tests determined whether these patterns of recidivism differed significantly between risk classification groups.

To estimate the predictive validity of the LSI–R:SV, LS/RNR and HCR–20^V3^, while accounting for varying times at risk in the community, Cox regression analyses (Cox, 1972) were used. A ‘time at risk’ (i.e. survival time) variable was created, with time at risk being calculated differently depending on when the risk assessment occurred. If the individual was assessed within the index incarceration, time at risk commenced from the index release date and ceased at either the first offence date (excluding any days of incarceration that occurred post index release) or the end of the follow-up period (31st December 2019). If the individual was assessed after their index incarceration but during a subsequent period of incarceration, time at risk commenced from the subsequent incarceration release date and ceased at either first offence (excluding any days of incarceration that may have occurred post release) or the end of the follow-up period. Finally, if the individual was assessed within the community (i.e. not within any incarceration period), time at risk commenced from the assessment date and ceased at either first offence (excluding any days of incarceration that may have occurred) or the end of the follow up. To determine the increase in the hazard of recidivating for a 1-unit increase in the predictor variable, hazard ratios (*e^B^*) were produced using the risk measure score. Where the proportional hazards assumption is violated, Struthers and Kalbfleisch (1986) suggest that the hazard ratio can be interpreted as the average hazard over time rather than a proportional hazard consistent over time. For models where these violations occurred, we utilised robust sandwich standard errors recommended as a method of determining significance where models are misspecified (Lin & Wei, 1989).

The receiver operating characteristic (ROC) curve was used to examine the ability of the risk measures to correctly classify participants into those who recidivated versus those who did not. The ROC curve plots the sensitivity against 1 – specificity at various thresholds and is independent of the base rate (Cook, 2007; Singh, 2013). The area under the curve (AUC) in a ROC analysis indicates the probability that a randomly selected recidivist would receive a higher risk score on the risk measure than a randomly selected individual who did not recidivate (Singh, 2013; Swets et al., 2000). The AUC Concordance Index (*C*-Index) value and time-dependent AUCs at five time-at-risk intervals (30, 90, 180, 360 and 720 days) were calculated for risk assessment scores for each recidivism outcome (any, violent and non-violent recidivism). The *C*-Index produces an overall assessment of the discriminant power of the model, with a value of .5 indicating chance-level discrimination and a value of 1.0 indicating perfect discrimination. Although no universal categorisation for the interpretation of AUCs exists, in forensic psychology and criminology research, AUC values are generally interpreted as .56–.63 = small effect, .64–.70 = medium effect, and .71 and above as a large effect (Rice & Harris, 2005).

Next, integrated Brier scores (IBS) were calculated by subtracting a risk measure’s predicted probability of offending from the actual outcome (either 0 for no recidivism or 1 for recidivism in this case), squaring this result and then averaging across all participants. Where multiple Brier scores can be calculated at different times, the integrated Brier score provides an overall assessment of the predictive power of the model across all available time-points. To help interpret the IBS, we calculated the Brier skill score (BSS; Wilks, 1995), which compares the predictive accuracy of one model to another. In this case, we compared the predictive accuracy of the risk assessment measure to a model that used a coin toss to ascertain risk (i.e. 50% probability of recidivism). See Supplemental Material for the BSS calculations. The BSS ranges from –∞ to 1, with a score of 0 suggesting that the risk measure predicts recidivism equal to a coin toss, while a positive BSS would suggest that the risk measure predicts recidivism better than a coin toss, with a BSS closer to 1 suggesting better prediction.

Finally, sensitivity, specificity, positive predictive values (PPV) and negative predictive values (NPV) were calculated to examine the discriminative performance of the LSI–R:SV, LS/RNR and HCR–20^V3^. Variations in sensitivity, specificity, PPV and NPV values result across differing risk assessment cut-off thresholds and at various follow-up periods. These statistics were therefore computed and reported across varying cut-off thresholds at 360 days at risk. For the LS measures, cut-off scores were chosen to correspond with risk categories. For the HCR–20^V3^, we examined the structured professional judgement ratings and total score using a cut score of 29 or below as this cut-off has been used widely within the HCR–20 research.

We did not examine the discriminative and predictive validity of the risk assessment measures separately for Aboriginal and Torres Strait Islander women and non-Aboriginal and Torres Strait Islander women. This was due to the small number of Aboriginal and Torres Strait Islander women included in the sample (see below) and is an important limitation of our analysis.

## Results

### Descriptive analysis

Among the 79 women in the sample, 17 (21.5%) identified as Aboriginal and Torres Strait Islander Peoples. The mean age of the participants at the time of their risk assessment was 33.22 years (*SD* = 7.64; range = 20–51) for the LSI–R:SV sample, 34.10 years (*SD* = 7.96; range = 20–52) for the LS/RNR sample, and 33.04 years (*SD* = 7.66; range = 21–52) for the HCR–20^V3^ sample. Most risk assessments were administered during participants’ time in prison, as opposed to time on a community corrections or parole order (see Table 1). The mean follow-up time from the beginning of the at-risk period to either first charge (for recidivists) or the end of the observation period (for non-recidivists) was 342.92 days (*SD* = 374.96; range = 4–1616) for the LSI–R:SV sample, 333.55 days (*SD* = 374.43; range = 3–1581) for the LS/RNR sample and 336.63 days (*SD* = 321.78; range = 2–1215) for the HCR–20^V3^ sample.

**Table 1.** Time between when the assessments were conducted relative to release from prison.

Assessment	Days between assessment and release from prison^**a**^			Days between release from prison and assessment^b^		
	*n*	*M* (*SD*)	Range	*n*	*M* (*SD*)	Range
LSI–R:SV	72	383.06 (339.78)	0–1337	4	232.5 (72.02)	161–296
LS/RNR	54	207.09 (236.63)	7–1106	24	119.17 (135.61)	9–514
HCR–20^V3^	42	391.86 (253.99)	41–1110	12	310.83 (232.43)	43–664

Note: LSI–R:SV = Level of Service Inventory–Revised: Screening Version; LS/RNR = Level of Service/Risk, Need, Responsivity; HCR–20^V3^ = Historical, Clinical, Risk Management 20–Version 3.

^a^Conducted in prison. ^b^Conducted in community.

Table 2 describes participants’ scores on the risk assessment measures. LSI–R:SV total scores ranged from 1 to 8 (*M =* 5.80, *SD* = 1.62), LS/RNR total scores ranged from 14 to 43 (*M* = 28.40, *SD* = 7.55), and HCR–20^V3^ total scores ranged from 15 to 39 (*M* = 25.98, *SD* = 5.92). Very few women were identified as having an overall low level of risk/need according to the three measures. This likely reflects the nature of the sample – that is, imprisoned women convicted of a serious violent offence – whereby increased complexity and/or seriousness of offending is expected relative to the broader population of justice-involved women.

**Table 2.** Descriptive statistics of the LSI–R:SV, LS/RNR and HCR–20^V3^.

Measure	*n*	*M*	*SD*	Low	Medium	High
				*n* (%)	*n* (%)	*n* (%)
LSI–R:SV	76	5.80	1.62	3 (3.9)	22 (28.9)	51 (67.1)
LS/RNR Total	78	28.40	7.55	0 (0)	13 (16.7)	65 (83.3)
Criminal history	78	6.35	1.73	7 (9.0)	6 (7.7)	65 (83.3)
Education / employment	78	5.72	2.33	17 (21.8)	16 (20.5)	45 (57.7)
Family / marital	78	2.54	1.38	21 (26.9)	10 (12.8)	47 (60.3)
Leisure / recreation	78	1.63	0.65	7 (9.0)	15 (19.2)	56 (71.8)
Antisocial companions	78	3.33	0.96	5 (6.4)	12 (15.4)	61 (78.2)
Alcohol & drug problem	78	4.88	2.28	19 (24.4)	9 (11.5)	50 (64.1)
Procriminal attitude orientation	78	1.96	1.38	27 (34.6)	21 (26.9)	30 (38.5)
Antisocial pattern	78	1.99	1.22	27 (34.6)	20 (25.6)	31 (39.7)
HCR–20^V3^ total	54/47^a^	25.98	5.92	1 (1.9)	24 (44.4)	29 (53.7)
H-scale	65	15.90	2.61	–	–	–
C-scale	47	3.91	2.33	–	–	–
R-scale	47	6.05	2.74	–	–	–

Note: LSI–R:SV = Level of Service Inventory–Revised: Screening Version; LS/RNR = Level of Service/Risk, Need, Responsivity; HCR–20^V3^ = Historical, Clinical, Risk Management 20–Version 3.

^a^*n* = 54 for structured professional judgement risk-level (low, moderate, high) data; *n* = 47 for total score item-level (*M, SD*) data.

Table 3 describes the base rates of recidivism and days to recidivism in the total sample, and separately for each risk measure sub-sample. Overall, almost two thirds (*n* = 49; 62.0%) of the sample received further police charges, with one in three (*n* = 29, 36.7%) recidivating violently and over half (*n* = 46, 58.2%) recidivating non-violently. For illustrative purposes, see Supplementary Materials for a figure indicating the survival probabilities (i.e. probability of not recidivating) for any, violent and non-violent recidivism in the largest risk assessment sample, the LS/RNR sample.

**Table 3.** Recidivism outcomes by total sample and risk measure sub-sample.

	Sample			
	Total (*N* = 79)	LSI–R:SV (*n* = 76)	LS/RNR (*n* = 78)	HCR–20^V^ (*n* = 54)
*Rate of recidivism*	*n* (%)	*n* (%)	*n* (%)	*n* (%)
Any recidivism	49 (62.0)	48 (63.2)	48 (61.5)	23 (42.6)
Violent recidivism	29 (36.7)	29 (38.2)	28 (35.9)	12 (22.2)
Non-violent recidivism	46 (58.2)	45 (59.2)	46 (59.0)	22 (40.7)
*Time (days) at risk to recidivism*		*M* (*SD*)	*M* (*SD*)	*M* (*SD*)
Any recidivism	—	221.75 (241.48)	187.08 (218.99)	207.43 (223.97)
Violent recidivism	—	241.21 (219.19)	215.46 (217.22)	204.00 (238.59)
Non-violent recidivism	—	222.84 (245.96)	190.87 (221.09)	218.05 (223.51)

Note: LSI–R:SV = Level of Service Inventory–Revised: Screening Version; LS/RNR = Level of Service/Risk, Need, Responsivity; HCR–20^V3^ = Historical, Clinical, Risk Management 20–Version 3.

### Validity of the risk/need measures for women

**Figure 1.**
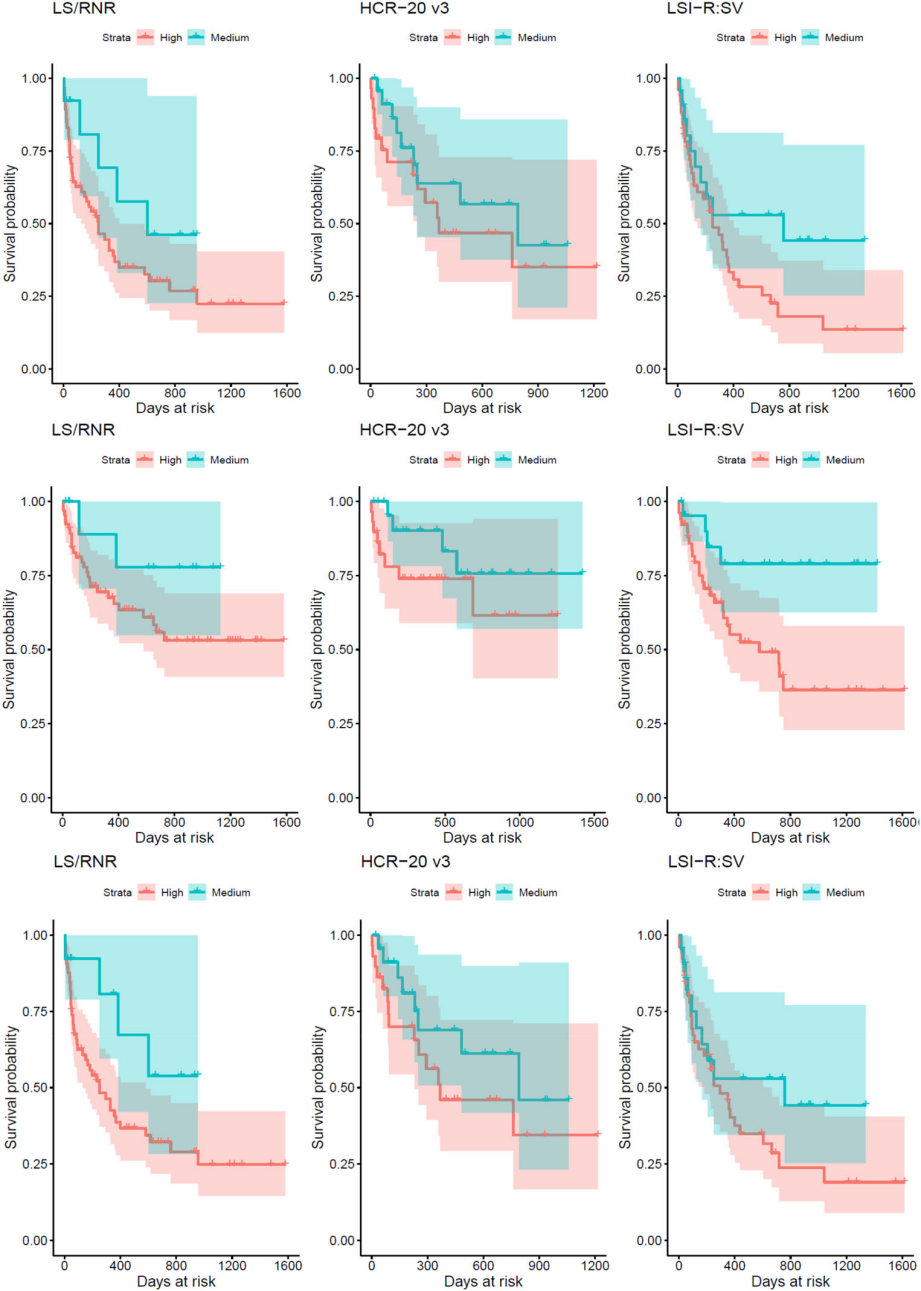
*Surv*ival curves *in the LSI-R:SV sample, LS/RNR* sample*, and HCR-20^V3^* sample depicting the probability of surviving (i.e., not recidivating) for any (top), violent (middle), and non-violent (bottom) recidivism according to risk classification.

Table 4 shows the proportion of the sample who recidivated while at risk in the community according to the risk classification for each measure. In all instances, rates of recidivism were higher among individuals classified as high risk than among those classified as medium risk. Kaplan–Meier survival analyses were conducted to visually observe the differences in survival probabilities across risk level groups (see Figure 1). Due to small samples, individuals classified as low risk were not included in these survival analyses. For all outcomes, the survival curves demonstrated that the probability of recidivism was greater for individuals classified as high risk on the three measures than for individuals classified as medium risk. Log-rank tests indicated that significant differences between survival curves emerged only for the LSI–R:SV sample with respect to violent recidivism, with the medium risk group having a significantly larger proportion survive (i.e. not reoffend) by the end of follow up, χ^2^(1) = 5.9, *p* = .02.

**Table 4.** Recidivism Outcomes by Risk Level on the LSI–R:SV, LS/RNR and HCR–20^V3^.

	Recidivism		
	Any	Violent	Non-violent
Risk level	*n* (%)	*n* (%)	*n* (%)
LSI–R:SV			
Low	2 (66.7)	0 (0)	2 (66.7)
Medium	10 (45.5)	4 (18.2)	10 (45.5)
High	36 (70.6)	25 (49.0)	33 (64.7)
LS/RNR			
Low	—	—	—
Medium	5 (38.5)	2 (15.4)	4 (30.8)
High	43 (66.2)	26 (40.0)	42 (64.6)
HCR–20^V3^			
Low	0 (0)	0 (0)	0 (0)
Moderate	9 (37.5)	4 (16.7)	8 (33.3)
High	14 (48.3)	8 (27.6)	14 (48.3)

Note: LSI–R:SV = Level of Service Inventory–Revised: Screening Version; LS/RNR = Level of Service/Risk, Need, Responsivity; HCR–20^V3^ = Historical, Clinical, Risk Management 20–Version 3.

A series of Cox regressions were performed to assess the ability of the LSI–R:SV, LS/RNR and HCR–20^V3^ to predict recidivism while accounting for varying times at risk to the community. The proportional hazards assumption was met in all cases, except where otherwise stated. The *C*-Index and the BSS were also computed to measure cumulative discrimination and calibration, respectively.

#### LS measures

Table 5 displays the Cox regression analyses for the total scores on the LSI–R:SV and LS/RNR, as well as the subscales of the LS/RNR. The proportional hazards assumption was violated for the Criminal History domain/any recidivism model and Leisure and Recreation domain/violent recidivism model (see Statistical Analysis for impact on interpretation). The results suggested that the LSI–R:SV score was only significant in the violent recidivism model, with the *C*-Index indicating small cumulative discrimination ability. The time-dependent AUCs at five time-at-risk intervals (30, 90, 180, 360 and 720 days) ranged from .61 (90 days) to .71 (720 days) for violent recidivism. However, the BSS suggested that the LSI–R:SV score demonstrated predictive validity for all recidivism outcomes, even though not all outcomes evidenced discriminative validity. This suggests that while the scores on the LSI–R:SV may have had a small relationship with the probability of each recidivism outcome (i.e. predictive validity), those who recidivated may not have received higher scores on the LSI–R:SV (i.e. discriminative validity). Indeed, looking at the rates of recidivism in Table 4, it appears that a similar proportion of individuals in the low- and high-risk categories recidivated, which likely affected the discriminative validity.

**Table 5.** Cox regression survival analysis and AUCs of the LSI–R:SV, LS/RNR, total scores and LS/RNR subscales for recidivism outcomes.

Sample	*n*	Recidivism		
		Any	Violent	Non-violent
LSI–R:SV total score	76			
HR [95% CI]		1.19 [0.98, 1.44]	1.44** [1.09, 1.90]	1.15 [0.95, 1.39]
*C*-Index^a^		.55	.63	.53
AUC at 30 days		.65	.66	.55
AUC at 90 days		.56	.61	.55
AUC at 180 days		.52	.65	.52
AUC at 360 days		.64	.69	.61
AUC at 720 days		.77	.71	.75
IBS^a^		.18	.20	.20
Brier skill score		0.33	0.24	0.23
LS/RNR total score	78			
HR [95% CI]		1.05* [1.01, 1.10]	1.09** [1.03, 1.15]	1.06** [1.02, 1.11]
*C*-Index^a^		.61	.67	.61
AUC at 30 days		.67	.71	.57
AUC at 90 days		.65	.73	.63
AUC at 180 days		.65	.75	.69
AUC at 360 days		.66	.77	.69
AUC at 720 days		.72	.72	.76
IBS^a^		.18	.17	.18
Brier skill score		0.32	0.37	0.30
	*Criminogenic needs according to the LS/RNR*			
Criminal history	78			
HR [95% CI]		1.32* [1.05, 1.66]	1.96** [1.29, 2.98]	1.31* [1.03, 1.68]
*C*-Index^a^		.63	.68	.62
AUC at 30 days		.66	.71	.56
AUC at 90 days		.68	.67	.67
AUC at 180 days		.68	.71	.68
AUC at 360 days		.70	.77	.72
AUC at 720 days		.67	.76	.69
IBS^a^		.18	.17	.19
Brier skill score		0.32	0.37	0.26
Education and employment	78			
HR [95% CI]		1.10 [0.96, 1.25]	1.15 [0.97, 1.36]	1.13 [1.00, 1.29]
*C*-Index^a^		.58	.58	.59
AUC at 30 days		.68	.65	.60
AUC at 90 days		.62	.60	.60
AUC at 180 days		.62	.63	.66
AUC at 360 days		.61	.60	.65
AUC at 720 days		.61	.60	.65
IBS^a^		.19	.21	.20
Brier skill score		0.28	0.22	0.23
Family and marital	78			
HR [95% CI]		1.08 [0.87, 1.33]	1.42* [1.04, 1.94]	1.11 [0.89, 1.39]
*C*-Index^a^		.55	.63	.55
AUC at 30 days		.61	.62	.51
AUC at 90 days		.59	.71	.57
AUC at 180 days		.59	.69	.60
AUC at 360 days		.57	.67	.59
AUC at 720 days		.65	.70	.67
IBS^a^		.19	.20	.20
Brier skill score		0.28	0.26	0.23
Leisure and recreation	78			
HR [95% CI]		1.51 [0.92, 2.50]	2.02 [0.98, 4.17]	1.53 [0.91, 2.57]
*C*-Index^a^		.58	.61	.59
AUC at 30 days		.66	.65	.65
AUC at 90 days		.66	.68	.66
AUC at 180 days		.64	.66	.66
AUC at 360 days		.61	.65	.60
AUC at 720 days		.55	.55	.54
IBS^a^		.18	.20	.20
Brier skill score		0.32	0.26	0.23
Antisocial companions	78			
HR [95% CI]		0.91 [0.69, 1.21]	1.11 [0.75, 1.63]	0.95 [0.71, 1.28]
*C*-Index^a^		.52	.52	.50
AUC at 30 days		.43	.39	.38
AUC at 90 days		.50	.52	.49
AUC at 180 days		.52	.55	.54
AUC at 360 days		.58	.63	.60
AUC at 720 days		.43	.50	.46
IBS^a^		.19	.21	.20
Brier skill score		0.28	0.22	0.23
Alcohol and drug problem	78			
HR [95% CI]		1. 16* [1.01, 1.32]	1.21* [1.00, 1.46]	1.18* [1.03, 1.35]
*C*-Index^a^		.58	.63	.59
AUC at 30 days		.62	.59	.64
AUC at 90 days		.61	.62	.62
AUC at 180 days		.61	.65	.63
AUC at 360 days		.58	.68	.59
AUC at 720 days		.70	.63	.69
IBS^a^		.19	.20	.19
Brier skill score		0.28	0.26	0.26
	Procriminal attitude orientation			
HR [95% CI]	78	1.12 [0.90, 1.38]	1.24 [0.95, 1.64]	1.16 [0.93, 1.44]
*C*-Index^a^		.54	.59	.54
AUC at 30 days		.59	.70	.48
AUC at 90 days		.56	.66	.54
AUC at 180 days		.56	.63	.57
AUC at 360 days		.56	.65	.59
AUC at 720 days		.73	.66	.76
IBS^a^		.18	.19	.19
Brier skill score		0.32	0.30	0.26
Antisocial pattern				
HR [95% CI]	78	1.15 [0.91, 1.46]	1.41* [1.03, 1.93]	1.22 [0.95, 1.55]
*C*-Index^a^		.55	.62	.55
AUC at 30 days		.60	.65	.51
AUC at 90 days		.56	.65	.54
AUC at 180 days		.58	.69	.59
AUC at 360 days		.57	.70	.61
AUC at 720 days		.68	.69	.71
IBS^a^		.18	.19	.18
Brier skill score		0.32	0.30	0.30

Note: LSI–R:SV = Level of Service Inventory–Revised: Screening Version; LS/RNR = Level of Service/Risk, Need, Responsivity; AUC = area under the curve; HR = hazard ratio; CI = confidence interval; IBS = integrated Brier score.

^a^Bootstrapping validation (with 300 bootstrapped datasets) indicated that bootstrap *C*-Index and bootstrap IBS values were within 0.03 and 0.05 of reported *C*-Index and reported IBS values, respectively.

*p < .05. **p < .01.

The LS/RNR total scores were significant in the any, violent and non-violent recidivism models. The *C*-Index values suggested a small discrimination effect for any and non-violent recidivism and a moderate effect for violent recidivism. The time-dependent AUCs for any recidivism ranged from .65 (90 and 180 days) to .72 (720 days). For violent recidivism, AUCs ranged from .71 (30 days) to .77 (360 days) suggesting moderate-to-large discrimination; for non-violent recidivism, they ranged from .57 (30 days) to .76 (720 days). The BSS suggested that the LS/RNR total scores were better at predicting violent recidivism than any or non-violent recidivism among the sample.

When considering the subscales of the LS/RNR indicating criminogenic needs, only the Criminal History and Alcohol and Drug Problem subscales were significantly related to all recidivism outcomes. Both these subscales demonstrated relatively better discrimination for violent recidivism (*C-*index = .68 and .63, respectively) than any and non-violent recidivism; predictive validity was evidenced for all recidivism outcomes (BSS = 0.26 to 0.37 and 0.26 to 0.28, respectively). The Family/Marital and Antisocial Pattern subscales were also related to violent recidivism but were not related to any or non-violent recidivism. Both subscales demonstrated a small discriminative (*C-*index = .63 and .62, respectively) and positive predictive (BSS = 0.26 and 0.30, respectively) relationship with violent recidivism.

#### HCR–20^V3^

As can be seen in Table 6, the HCR–20^V3^ total score was not significantly related to any of the recidivism outcomes in the sample and demonstrated negligible discriminative validity (*C-*Index = 0.57) for violent recidivism, despite demonstrating strong predictive validity (BSS = 0.45). As there was often a substantial lag between the date of HCR–20^V3^ assessment and the date of release from prison, post hoc Cox regression analyses were run using the H-Scale score (static factors) alone (*n* = 65). The results indicated that the H-Scale scores were related to violent recidivism, demonstrating moderate cumulative discrimination (*C*-Index = .65) and relatively strong prediction (BSS = 0.40). The time-dependent AUCs for violent recidivism were larger at 30 and 90 days (.77 and .83, respectively) than at 180, 360 and 720 days (.63 to .64). The BSS suggests that the H-Scale scores, like the HCR–20^V3^ scores, demonstrated relatively greater predictive validity (BSS = 0.40) for violent recidivism than the LS assessments.

**Table 6.** Cox regression survival analysis and AUCs of the HCR–20^V3^ total score and H-Scale score for all recidivism outcomes.

Sample	*n*	Recidivism		
		Any	Violent	Non-violent
HCR–20^V3^ total score	54			
HR [95% CI]		0.99 [0.91–1.08]	1.01 [0.91–1.14]	1.01 [0.93–1.10]
*C*-Index^a^		.49	.57	.53
AUC at 30 days		.56	.56	.59
AUC at 90 days		.45	.53	.45
AUC at 180 days		.43	.50	.49
AUC at 360 days		.47	.57	.49
AUC at 720 days		.32	.48	.33
IBS^a^		.20	.18	.20
Brier skill score		0.22	0.45	0.23
*Post* *hoc analyses*				
HCR–20^V3^ H-Scale	65			
HR [95% CI]		1.05 [0.92–1.19]	1.26* [1.02–1.56]	1.04 [0.90–1.19]
*C*-Index^a^		.57	.65	.56
AUC at 30 days		.63	.77	.58
AUC at 90 days		.62	.83	.62
AUC at 180 days		.58	.63	.59
AUC at 360 days		.57	.63	.56
AUC at 720 days		.48	.64	.48
IBS^a^		.22	.18	.22
Brier skill score		0.12	0.40	0.12

Note: HCR–20^V3^ = Historical, Clinical, Risk Management 20–Version 3. AUC = area under the curve; HR = hazard ratio; CI = confidence interval; IBS = integrated Brier score.

^a^Bootstrapping validation (with 300 bootstrapped datasets) indicated that bootstrap *C*-Index and bootstrap IBS values were within 0.06 and 0.05 of reported *C*-Index and reported IBS values, respectively.

*p < .05.

### Sensitivity, specificity, PPV, NPV

Finally, sensitivity, specificity, PPV and NPV were calculated to further illustrate the discriminative and predictive performance of the LSI–R:SV, LS/RNR and HCR–20^V3^. Table 7 presents the test statistics for each assessment measure and recidivism outcomes at 360 days at risk.

**Table 7.** Sensitivity, specificity, positive predictive value and negative predictive value metrics for the measures at different cut-offs at 360 days at risk.

Cut-off	Recidivism											
	Any				Violent				Non-violent			
	Sens.	Spec.	PPV	NPV	Sens.	Spec.	PPV	NPV	Sens.	Spec.	PPV	NPV
LSI–R:SV^a^												
≤5	74.82	44.00	64.56	56.16	82.57	43.24	43.01	82.70	72.68	40.74	58.05	56.92
LS/RNR^a^												
≤19	92.29	23.08	61.34	69.36	95.18	19.51	33.10	90.64	94.59	22.22	58.85	77.73
≤29	57.22	73.08	73.76	56.36	76.44	73.17	54.38	88.13	60.80	74.07	73.39	61.64
HCR–20^V3^												
Mod: High	62.27	50.00	48.16	63.98	76.21	51.85	26.75	90.43	66.31	50.00	47.87	68.18
≤29	31.37	70.59	43.31	58.94	40.88	78.26	28.04	86.47	34.20	70.59	42.94	62.38
H-Scale												
16^b^	60.60	65.22	63.66	62.21	68.63	58.82	33.07	86.35	61.52	62.50	58.76	65.16

Note: LSI–R:SV = Level of Service Inventory–Revised: Screening Version; LS/RNR = Level of Service/Risk, Need, Responsivity; HCR–20^V3^ = Historical, Clinical, Risk Management 20–Version 3. Sens. = sensitivity; Spec. = specificity; PPV = positive predictive value; NPV = negative predictive value; Mod = moderate.

^a^Cut-off scores corresponding to low risk are not included due to few/no women assessed as low risk. ^b^A cut-off score of 16 or below was used to explore test statistics for the H-Scale, reflecting the sample mean.

For the LS measures, sensitivity (i.e. the proportion of recidivists who were above the cut-off) was consistently higher than specificity (i.e. the proportion of non-recidivists who were at or below the cut-off) for violent recidivism, but not for any or non-violent recidivism. PPV values (i.e. the proportion of individuals above the cut-off who recidivated) suggested that the LS measures were generally more accurate in predicting any recidivism than violent recidivism; for violent recidivism, the measures had stronger predictive power when it came to predicting who would not recidivate (NPV values).

For all recidivism outcomes, the HCR–20^V3^ demonstrated higher sensitivity with the structured professional judgement ratings (i.e. moderate vs. high risk) than with the actuarial cut-off score. However, the actuarial score correctly identified a higher proportion of non-recidivists as being at or below the cut-off (specificity) than the moderate versus high-risk judgements. In all instances, the HCR–20^V3^ was less accurate when predicting who would recidivate (PPV) than predicting who would not recidivate (NPV). The H-Scale generally performed as well as, if not better than, the structured professional judgement ratings and the actuarial cut-off score for the HCR–20^V3^ as a whole.

## Discussion

This study explored the discriminative and predictive validity of the LSI–R:SV, LS/RNR and HCR–20^V3^ in a sample of Australian women sentenced to prison for serious violent offending. Results revealed that the LS/RNR was related to any, violent and non-violent recidivism, whereas the LSI–R:SV was only related to violent recidivism. According to the LS/RNR, criminal history, family and marital, alcohol and drug problem and antisocial pattern were the only needs related to recidivism for women in the sample. Although the H-Scale of the HCR–20^V3^ was related to violent recidivism, the HCR–20^V3^, as a whole, was not significantly related to recidivism despite displaying strong predictive validity for violent recidivism. It is likely that the HCR–20^V3^ was not significant in the Cox regression models due to the small sample size and the length of time between when the assessment was completed and when the participants were released from prison. As such, the dynamic variables on the C and R scales completed at the time of administration were unlikely to have been a valid measure of the item and scale scores at the time of release from custody. By contrast, the static H-scale items would have remained unchanged from the time of administration to the time at release.

### Risk assessment for female offenders

This study adds to a small body of evidence regarding the use of the LSI–R:SV with women. In contrast to previous research (Lowenkamp et al., 2009; McCafferty & Scherer, 2017), the LSI–R:SV was not related to any recidivism in women. However, it was related to violent recidivism, despite that not being the core intention of the risk assessment measure. This may be attributed to the unique characteristics of the small sample. As the present study only involved women convicted for a serious violent offence, it is unsurprising that very few participants were rated as low risk (i.e. *n* = 3). None of the low-risk individuals violently recidivated, which would have improved the predictive and discriminative validity of the LSI–R:SV for violence. However, two out of the three participants rated as low risk reoffended generally, which resulted in a greater proportion of recidivists in the low-risk group than in the medium-risk group. Due to the small sample size, two participants being misclassified significantly impacted the accuracy of the risk measure. As such, conclusions regarding the validity of the LSI–R:SV for use with females should be drawn cautiously.

In contrast, the full LS/RNR was related to all recidivism outcomes for women, demonstrating relatively strong discrimination, prediction, sensitivity, specificity and negative predictive power for violent recidivism. The LS/RNR demonstrated similar discriminative validity to other research on the use of LS measures with imprisoned females in Australia, which found that the LSI–R was moderately related to reincarceration (Watkins, 2011). In contrast, research examining the use of the LS/CMI with women in Tasmania serving community-based orders found that the LS/CMI was not related to recidivism within 12 months of their index incident (Gordon et al., 2015). It may be that the LS measures are less effective among lower risk women than in higher risk women such as those in the current sample or those who have been imprisoned.

Although the HCR–20^V3^ was not related to any of the recidivism outcomes, this may be due to the smaller sample size and low base rate of reoffending, as only 12 individuals recidivated in the HCR–20^V3^ sample. Although the scores did not discriminate between recidivists and non-recidivists, the HCR–20^V3^ scores were related to the probability of offending (i.e. demonstrated predictive validity). Again, these results may be attributed to the small sample size and relatively low base rate, making the findings unstable. It is also likely that the results were influenced by the period between assessment and release from prison, as the dynamic variables on the C-Scale and R-Scale will have likely fluctuated and do not accurately represent the participants’ risk at the time of release. To help account for this, we explored the relationship between the H-Scale and recidivism, as the number of participants who were assessed using only the H-Scale was greater, and the H-Scale does not fluctuate across time. Results revealed that the H-Scale demonstrated moderate discriminative validity and relatively strong predictive validity with violent recidivism. These findings are in line with Rossdale et al.’s (2020) meta-analysis, which found that the H-Scale was moderately related to recidivism among women and was the best predictor when considering the subscales and total score. Notably, this is the first study in Australia to explore the HCR–20^V3^ among women and the third study internationally to examine the HCR–20^V3^ in females who were imprisoned. Despite the small sample size, the findings are positive, suggesting that it may be a useful measure to identify females at risk of future violent recidivism; however, more research is needed.

### Needs for female serious violent offenders

This study contributes to a growing body of research exploring the generalisability of risk assessments for women (see Gower et al., 2020). Consistent with past research on the criminogenic needs as identified on the LS/RNR (Olver et al., 2014), this study found that not all subscales were related to recidivism for previously imprisoned females. Criminal history, family and marital problems, alcohol and drug problems and antisocial pattern of behaviour were the only criminogenic needs that were significantly related to recidivism. However, remaining needs (education and employment, leisure and recreation, antisocial companions and procriminal attitude orientation) demonstrated reasonable predictive validity for all recidivism outcomes, despite non-significant results in Cox regression models. It has been suggested that certain risk factors may be more relevant for women, thus indicating a gender-responsive approach to assessment, treatment and management (Brennan et al., 2012; Hannah-Moffat, 2009; Salisbury et al., 2016). For example, prior work has found that family/marital difficulties and substance use problems (in addition to economic disadvantage and emotional concerns) may have special relevance for some women’s pathways to offending (Andrews et al., 2012; Olver et al., 2014; Van Voorhis et al., 2010), which are often theorised to stem from experiences of abuse and victimisation (Salisbury et al., 2016). However, it is difficult to draw conclusions from our data about either (a) the relative importance of criminogenic needs among women or (b) gender differences in needs, because of the small, unique sample and lack of gender comparisons.

## Limitations and future directions

Although investigating the validity of common risk assessment measures among a high-risk sample of women is important, the small sample and restricted variability in risk scores limit the generalisability and power of the findings. It also meant we could not examine potential cross-cultural differences in the validity of these measures for women, which requires greater attention in future research. Further, the imperfect nature of real-world data collection meant that not all serious violent offenders in Victoria were assessed using each of the risk measures. As such, non-mutually exclusive samples were used to validate each risk measure in this study. Further, the assessments varied in terms of when they were conducted relative to the participants’ release dates, and we could not account for the potential impact of treatment given the small sample (*n* = 45 women had a treatment participation during the study period) and variation in treatments received. Nevertheless, highly controlled validation studies (e.g. involving highly trained researchers scoring risk assessment measures with high levels of inter-rater reliability) indicate how well measures *can* perform, whereas real-world studies like this indicate how well they *do* perform in the clinical/correctional context in which they are used (Edens & Boccaccini, 2017). Our findings that the LS/RNR was related to all recidivism outcomes and that the HCR–20^V3^ H-Scale was related to violent recidivism highlight the robustness of these measures in samples of women (regardless of the limitations of the small sample size and use of field data).

The validity of a risk assessment at a single time-point is useful to know but does not reflect the realities of how risk assessments are often used in practice. Within the Corrections Victoria context, the HCR–20^V3^ is rarely readministered, but the LS/RNR is readministered regularly. However, this study did not explore the fluctuation in dynamic risk over time among women and whether those fluctuations were related to changes in recidivism. Increasingly, researchers have been examining the dynamic nature of risk and how reassessment of risk and protective factors over time is related to offending (see Babchishin & Hanson, 2020; Davies et al., 2022; Lloyd et al., 2020; Simmons et al., 2023). However, there is limited research exploring gender differences in the dynamic nature of risk assessment measures, which may be a useful avenue for future research.

## Conclusion

Overall, this research suggests that the LS/RNR and HCR–20^V3^ H-Scale are useful assessments for the prediction and discrimination of future offending for females sentenced to prison for a serious violent offence. Of the LS/RNR criminogenic needs domains, it appears that substance use and problems with relationships were important risk factors for females in the sample, in addition to a history of criminal and antisocial behaviour. Future research drawing upon larger samples and investigating how the needs identified on the risk assessment measures fluctuate over time may help advance the literature.

## Supplementary Material

Supplemental Material

## Footnotes

1 Examples (non-exhaustive) of such offences include causing serious injury intentionally or recklessly; threats to kill or inflict serious injury; kidnapping; false imprisonment; inflicting grievous bodily harm; armed robbery; aggravated burglary or home invasion; manslaughter; murder.
