# Supplementary material for: Discriminative and predictive validity of risk assessment measures for women incarcerated for serious violent offences in Australia
Source: Psychiatr Psychol Law. 2024 Jan 8;31(5):963–85. doi: 10.1080/13218719.2023.2242437 (PMC11418047; doi:10.1080/13218719.2023.2242437)
Supplement: Supplemental Material [file TPPL_A_2242437_SM3849.docx]

**Supplementary Material**

**Figure 1**

*Survival Curves in the LS/RNR Sample (n = 78) Depicting the Probability of Surviving (i.e., Not Recidivating) for Any, Violent, and Non-violent Recidivism.*

**
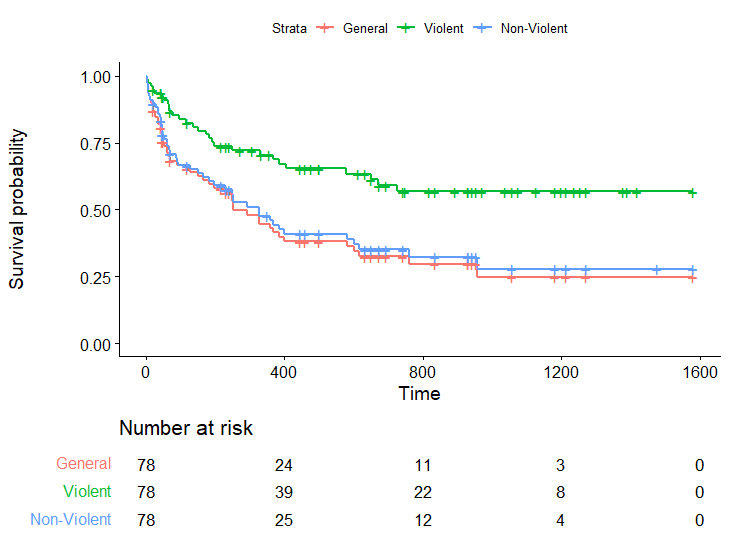
**

**Brier Skill Score Calculations**

The formula to calculate the Brier Score is

$$BS=\frac{1}{N}\sum_{t=1}^{N} {(f_{t}-o_{t})}^{2}$$

Where *f_t_* is the probability of the outcome (i.e., rate of recidivism) occurring and *o*_t_ is either a 1, if the outcome occurred or a 0, if the outcome did not occur.

To calculate the baseline prediction model (i.e., assuming a coin flip was used rather than a risk instrument to predict aggression), *f*_t_ was the rate of recidivism. We calculated the average squared probabilities by summing the difference between the probability of recidivism and 0 (outcome did not occur), and 1 (outcome occurred), then divided the sum by two (number of outcomes), and squared the result (See Table 1).

The formula to calculate the Brier Skill Score is

$BSS=1-\frac{BS}{{BS}_{ref}}$

Where *BS_ref_* is the Brier score of the baseline prediction that we are comparing against.

**Table 1**

*Calculated Integrated Brier Scores, Brier Scores, and Brier Skill Scores for Each Model*

| Instrument | Recidivism outcome | Rate of Recidivism | IBS | Baseline Brier Score | Brier Skill Score |
| --- | --- | --- | --- | --- | --- |
| LSI-R:SV | All | 0.63 | 0.18 | 0.27 | 0.33 |
|  | Violent | 0.38 | 0.20 | 0.26 | 0.24 |
|  | Nonviolent | 0.59 | 0.20 | 0.26 | 0.23 |
| LS/RNR | All | 0.62 | 0.18 | 0.26 | 0.32 |
|  | Violent | 0.36 | 0.17 | 0.27 | 0.37 |
|  | Nonviolent | 0.59 | 0.18 | 0.26 | 0.30 |
| HCR-20^V3^ | All | 0.43 | 0.20 | 0.25 | 0.22 |
|  | Violent | 0.22 | 0.18 | 0.33 | 0.45 |
|  | Nonviolent | 0.41 | 0.20 | 0.26 | 0.23 |
| HCR H-Scale | All | 0.51 | 0.22 | 0.25 | 0.12 |
|  | Violent | 0.28 | 0.18 | 0.30 | 0.40 |
|  | Nonviolent | 0.48 | 0.22 | 0.25 | 0.12 |
| LS/RNR - CH | All | 0.62 | 0.18 | 0.26 | 0.32 |
|  | Violent | 0.36 | 0.17 | 0.27 | 0.37 |
|  | Nonviolent | 0.59 | 0.19 | 0.26 | 0.26 |
| LS/RNR - EE | All | 0.62 | 0.19 | 0.26 | 0.28 |
|  | Violent | 0.36 | 0.21 | 0.27 | 0.22 |
|  | Nonviolent | 0.59 | 0.20 | 0.26 | 0.23 |
| LS/RNR - FM | All | 0.62 | 0.19 | 0.26 | 0.28 |
|  | Violent | 0.36 | 0.20 | 0.27 | 0.26 |
|  | Nonviolent | 0.59 | 0.20 | 0.26 | 0.23 |
| LS/RNR - LR | All | 0.62 | 0.18 | 0.26 | 0.32 |
|  | Violent | 0.36 | 0.20 | 0.27 | 0.26 |
|  | Nonviolent | 0.59 | 0.20 | 0.26 | 0.23 |
| LS/RNR - AC | All | 0.62 | 0.19 | 0.26 | 0.28 |
|  | Violent | 0.36 | 0.21 | 0.27 | 0.22 |
|  | Nonviolent | 0.59 | 0.20 | 0.26 | 0.23 |
| LS/RNR - ADP | All | 0.62 | 0.19 | 0.26 | 0.28 |
|  | Violent | 0.36 | 0.20 | 0.27 | 0.26 |
|  | Nonviolent | 0.59 | 0.19 | 0.26 | 0.26 |
| LS/RNR - PA | All | 0.62 | 0.18 | 0.26 | 0.32 |
|  | Violent | 0.36 | 0.19 | 0.27 | 0.30 |
|  | Nonviolent | 0.59 | 0.19 | 0.26 | 0.26 |
| LS/RNR - AP | All | 0.62 | 0.18 | 0.26 | 0.32 |
|  | Violent | 0.36 | 0.19 | 0.27 | 0.30 |
|  | Nonviolent | 0.59 | 0.18 | 0.26 | 0.30 |

*Note.* CH = criminal history; EE = education and employment; FM = family and marital; LR = leisure and recreation; AC = antisocial companions; ADP = alcoholg and drug problem; PA = procriminal attitude orientation; AP = antisocial pattern.
